# Apolipoprotein E in dense deposits: a parting of the ways in C3 glomerulopathy

**DOI:** 10.1093/ckj/sfae161

**Published:** 2024-06-03

**Authors:** Joana Tavares, Fernando Caravaca-Fontán, Manuel Praga

**Affiliations:** Department of Nephrology, Centro Hospitalar e Universitário do Porto, Porto, Portugal; Department of Nephrology, Instituto de Investigación Hospital 12 de Octubre, Madrid, Spain; Department of Medicine, Complutense University, Madrid, Spain

Formerly known as type II membranoproliferative glomerulonephritis, C3 glomerulopathy (C3G) is a glomerular disease characterized by C3 staining on immunofluorescence and results from dysregulation on the alternative pathway of complement. It comprises two subtypes: C3 glomerulonephritis (C3GN) and dense deposit disease (DDD). As the name suggests, DDD is distinguished by the presence of dense deposits along the glomerular basement membrane (GBM) on electron microscopy [1]. The pathophysiological mechanism of the formation of these deposits is not yet understood, but there appears to be a lipid component, as previous studies have shown that they are osmium dependent [2].

**Figure 1:**
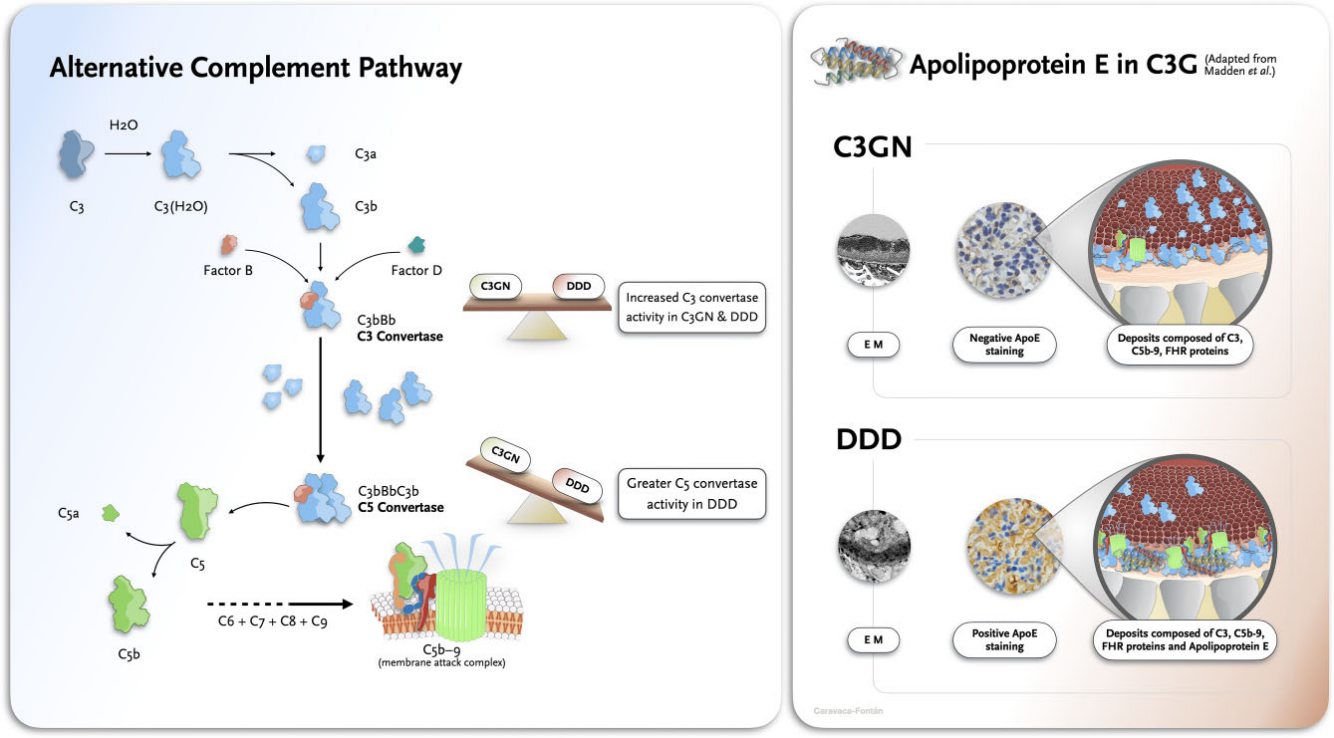
Insights on the pathophysiology of C3G. (Left panel) Alternative complement pathway. In the study by Madden *et al.* [3], the accumulation of C5-9 was more prominent in DDD, suggesting a greater C5 convertase activity. (Right panel) Electron microscopy findings and apoE staining in C3GN and DDD.

In a new cutting-edge study [3] conducted at the Mayo Clinic and led by Benjamin Madden and Sanjeev Sethi, who have been at the forefront of applying mass spectrometry to kidney biopsies, the researchers evaluated the major components of deposits in DDD compared with C3GN. They performed laser capture microdissection (LCM) of glomeruli followed by mass spectrometry (MS) in 12 patients each of DDD, C3GN and pretransplant kidney control biopsies. As expected, LCM-MS showed an accumulation of complement components, namely C3, C5, C6, C7, C8, C9, CFH, CFHR1 and CFHR5, as compared with controls. The accumulation of C3 and complement regulatory proteins was comparable between C3GN and DDD, suggesting an increased C3 convertase activity in both entities. Conversely, the accumulation of C5-9 was more prominent in DDD, suggesting a greater C5 convertase activity in this histologic subtype. The most remarkable finding was that DDD was enriched with apolipoprotein E (apoE) compared with C3GN and control cases. Blinded validation studies using apoE staining by immunohistochemistry confirmed the diagnosis of DDD and C3GN in 80 and 81.3% of patients, respectively. This suggests that apoE staining can be a useful alternative to electron microscopy, especially when this technique is not available (Fig. 1).

While this study marks a significant breakthrough in the diagnosis of the disease, facilitating the correct identification of this histologic subtype, critical gaps remain in the understanding of its biological implications. Beyond its role as a lipid transport protein, apoE has been implicated in various biological processes associated with other diseases (Table 1) [4]. These diseases commonly involve the formation of deposits, suggesting that apoE may function as a chaperone protein. Additionally, apoE is known to play a role in immunoregulation. Studies in animal models have demonstrated that it can inhibit inflammation by binding to the classical pathway activator C1q and complement factor H, thereby regulating the alternative pathway of complement [5].

**Table 1:** Diseases associated with apoE.

Kidney diseases	Other diseases
Fibrillary glomerulonephritis Immunotactoid glomerulonephritis Monoclonal immunoglobin deposition disease Lipoprotein glomerulopathy	Alzheimer's disease Amyloidosis Atherosclerosis Age-related macular degeneration

Thus, considering the fact that apoE has been identified as a component of drusen bodies in age-related macular degeneration, along with C5 and C5b-9 complex [6], the authors hypothesize that dense deposits could result from apoE binding to the GBM and acts as a chaperone protein for C5-9, especially when apoE levels are increased, as seen in DDD. Despite these groundbreaking findings, further research will be needed to elucidate the mechanisms by which apoE binds to C5-9 proteins and to understand the pathogenic role of serum apoE levels in DDD [3].
